# HGBEnviroScreen: Enabling Community Action through Data Integration in the Houston–Galveston–Brazoria Region

**DOI:** 10.3390/ijerph17041130

**Published:** 2020-02-11

**Authors:** Sharmila Bhandari, P. Grace Tee Lewis, Elena Craft, Skylar W. Marvel, David M. Reif, Weihsueh A. Chiu

**Affiliations:** 1 Veterinary Integrative Biosciences, College of Veterinary Medicine and Biomedical Sciences, Texas A&M University, College Station, TX 77845, USA; sbhandari@cvm.tamu.edu; 2 Environmental Defense Fund, 301 Congress Ave #1300, Austin, TX 78701, USA; glewis@edf.org (P.G.T.L.); ecraft@edf.org (E.C.); 3 Department of Biological Sciences, North Carolina State University, Raleigh, NC 27695, USA; swmarvel@ncsu.edu (S.W.M.); dmreif@ncsu.edu (D.M.R.)

**Keywords:** environmental justice, geographic information systems, vulnerable populations, community, public health, data, screening, enviroscreen

## Abstract

The Houston–Galveston–Brazoria (HGB) region faces numerous environmental and public health challenges from both natural disasters and industrial activity, but the historically disadvantaged communities most often impacted by such risks have limited ability to access and utilize big data for advocacy efforts. We developed HGBEnviroScreen to identify and prioritize regions of heightened vulnerability, in part to assist communities in understanding risk factors and developing environmental justice action plans. While similar in objectives to existing environmental justice tools, HGBEnviroScreen is unique in its ability to integrate and visualize national and local data to address regional concerns. For the 1090 census tracts in the HGB region, we accrued data into five domains: (i) social vulnerability, (ii) baseline health, (iii) environmental exposures and risks, (iv) environmental sources, and (v) flooding. We then integrated and visualized these data using the Toxicological Prioritization Index (ToxPi). We found that the highest vulnerability census tracts have multifactorial risk factors, with common drivers being flooding, social vulnerability, and proximity to environmental sources. Thus, HGBEnviroScreen is not only helping identify communities of greatest overall vulnerability but is also providing insights into which domains would most benefit from improved planning, policy, and action in order to reduce future vulnerability.

## 1. Introduction

While a strong economy that creates jobs and wealth is fundamental to society, the social and financial costs and benefits produced by economic growth are not shared equally. In particular, communities of color and low wealth have historically borne a disproportionate burden of the environmental costs of economic growth. In Houston, it was found in the 1980s that uncontrolled toxic waste sites were located in heavily over-represented African-American neighborhoods [1]. Thus, geospatial information about environmental burdens was a key factor in the birth of the environmental justice (EJ) movement, and the tenet that “all people and communities are entitled to equal protection of environmental and public health laws and regulations [2]”. 

The Houston area provides an exemplary illustration of the challenges of EJ. In addition to being among the most populous metropolitan areas in the United States, Houston is home to the largest petrochemical complex in North America and two of the largest refineries in the country. There are more than 400 chemical plants located along the Houston Ship Channel, which reaches within 5 miles of Downtown Houston [3]. It is also a large urban port city, with the Houston Ship Channel being a transportation hub for freight, further adding to regional air pollution. East Houston, where most of these facilities are concentrated, is predominantly populated by communities which are African-American and Hispanic and which suffer from poor environmental conditions resulting from not only routine industrial business practices but also natural and manmade disasters such as hurricanes and chemical tank fires. Moreover, due to historical segregation and redlining, many neighborhoods in East Houston lack deed restrictions, and the region as a whole has resisted land use zoning laws [4,5].

Overall, this situation presents a significant challenge for community-based organizations (CBOs) advocating for EJ. Many CBOs lack the political clout and scientific data needed to identify and address environmental and health inequities where they live, work, and go to school. The development of tools such as EJSCREEN by the U.S. EPA and CalEnviroScreen by the California Environmental Protection Agency (CalEPA), which incorporate indicators of pollution burden and vulnerability, is a step toward improving understanding of cumulative impacts on overburdened communities [6,7]. These tools use publicly available datasets to map spatial differences in environmental indicators that influence health. Visualizing this information using mapping provides the ability to identify and target where policy and government actions should be focused. However, given that EJSCREEN was developed using national-scale data, the value of the tool can be limited in understanding region-specific vulnerability indicators [8].

The objective of the work presented here was to develop an adaptable, community-based EJ screening tool for the Houston–Galveston–Brazoria (HGB) region that incorporates indicators reflecting the unique challenges of living with a large petrochemical industry and busy international ports. Designated “HGBEnviroScreen,” this tool also provides more region-specific data than are available through current tools such as EJSCREEN, allowing for the ability to understand additional factors that are driving vulnerability at a census tract level of resolution. In order to address the needs for prioritization of overall burden across communities as well as for prioritization of intervention opportunities within communities simultaneously, we adapted the Toxicological Prioritization Index (ToxPi) approach to integrate the multiple data streams incorporated into HGBEnviroScreen [9,10]. ToxPi is a visual, decision-support tool that has been used to communicate risk prioritization and profiling information between scientists, regulators, stakeholders, and the general public [11,12,13]. The overall ToxPi score and accompanying graphical profile simultaneously provide both a relative ranking in terms of overall vulnerability as well as an indicator of the specific drivers of increased vulnerability. The result of integrating ToxPi visualizations within the ArcGIS framework is an online, GIS-based, local screening tool. Below, we discuss the domains of vulnerability and corresponding indicators of concern in the HGB area, the scoring methodology and visualization tool, prioritization results, and its application in support of partner communities.

## 2. Materials and Methods

**Figure 1:**
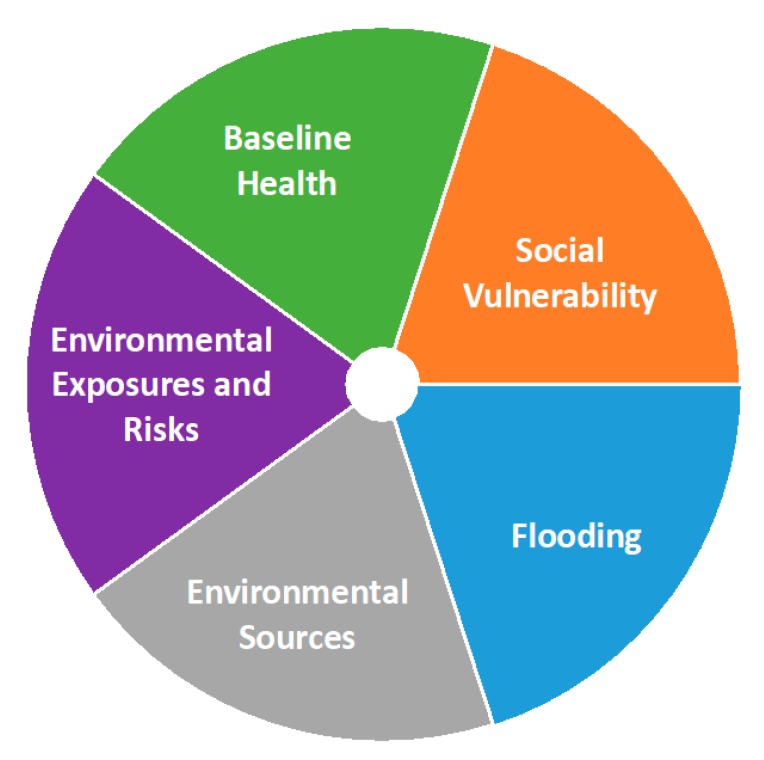
Domains of vulnerability of concern to partner communities in the Houston–Galveston–Brazoria (HGB) area (see Table 1 for details).

The aftermath of the August 2017 Hurricane Harvey clearly demonstrated discrepancies across Houston communities with regard to cumulative environmental impacts [14,15]. We gathered data at the census tract level for the eight-county HGB area, which consists of Brazoria, Chambers, Fort Bend, Harris, Galveston, Liberty, Montgomery, and Walker Counties. In total, the study area was comprised of 1090 census tracts. Publicly-available datasets providing indicators of environmental or health inequities were organized into five categories, or “domains”, with numerous indicators identified in each domain (see Figure 1 and Table 1), as follows:

1. Social Vulnerability, as described by the Center for Disease Control (CDC), “refers to the resilience of communities when confronted by external stresses on human health”, particularly with respect to natural or human-caused disasters or disease outbreaks, and typically consists of socioeconomic and demographic factors. Social vulnerability is relevant to environmental vulnerability in the HGB area because of the prevalence of disasters, both natural (e.g., flooding and hurricanes) and manmade (e.g., chemical spills, oil spills, and fires). We included the four main themes (socioeconomic status, household composition and disability, minority and language, and housing and transportation) in addition to one auxiliary indicator (percent without health insurance) from the CDC’s Social Vulnerability Index (SVI) in our analysis. We also added three indicators related to food/nutrition access and security. Existing EJ screening tools typically include individual indicators of socioeconomic status, but we used SVI both because it provides a more comprehensive portrait of social vulnerability, as well as because it has been widely used and validated [16].
2. Baseline Health uses indicators related to health status and health care access to capture the extent to which members of a community may be more vulnerable to effects from the environment, similar to the “sensitive population indicators” used in CalEnviroScreen [6]. Four of these indicators are of disease prevalence, specifically for coronary heart disease, stroke, childhood asthma, and chronic obstructive pulmonary disease, as estimated [17]. It is posited that higher rates of these diseases suggest a larger population that is more susceptible to the health impacts from environmental factors. Additionally, life expectancy is taken as an aggregate measure of baseline health, with lower values indicating greater vulnerability [18]. Finally, as a measure of access to health care, we counted the number of hospitals within 5 km of each census tract, again with lower numbers indicating greater vulnerability.
3. Environmental Exposures and Risks utilize three well-established sources of indicators of pollutant exposures and risk. First, the EPA Risk-Screening Environmental Indicators provide a screening-level metric for the potential for chronic human health risks due to toxic releases from facilities that report to the Toxics Release Inventory (TRI) [19]. Additionally, we utilized the most recently available U.S. EPA National Air Toxics Assessment calculations for cancer risk, respiratory effects, and reproductive effects [20]. Third, we included two separate estimates of PM2.5 concentrations: one based on the last three years available from the U.S. EPA’s Community Multiscale Air Quality (CMAQ) model, [21] and one based on satellite imagery [22]. Each of these indicators is available at the census tract level.
4. Environmental Sources are indicators related to the proximity of each census tract to sources of environmental emissions. Specifically, these indicators provide metrics for potential environmental exposure, rather than measured or predicted exposure or risk levels. A large number of different types of facilities were included. Point sources where exposure was expected to be more localized, such as Superfund sites and leaking petroleum storage tanks, were counted if located in each census tract. Industrial facilities such as cement batch plants and concrete crushers (combined), petroleum and oil refineries (combined), metal recyclers, and powerplants, where some transport of pollutants would be expected, were counted if within 1 km of each census tract. Additionally, proximity to major roads was also measured with a 1 km buffer distance. Finally, to incorporate indicators related to accident risks, facilities with registered Risk Management Plans were included using three metrics: facility counts, number of accidents in the five years before 30 April 2018, and total number of shelter-in-place events in the five years before 30 April 2018. These facilities were counted if located in each census tract.
5. Flooding reflects risks related to inundation by floodwaters and includes both frequency and severity metrics. The recent history of frequent flooding in the HGB area emphasizes the importance of this domain to evaluating environmental vulnerability. The fractions of the census tract area within 100- and 500-year FEMA flood plains were used as frequency indicators. For severity, the number of FEMA damage assessments at various levels (minimal, major, affected, and destroyed), as well as the percentage of households filing damage claims, were used as indicators.

The data were combined into an overall ToxPi model, as per the breakdown given in Table 1. In ToxPi model terms, each data slice (e.g., “Socioeconomic Status”) was assigned to an appropriate domain (e.g., “Social Vulnerability”), where each domain is composed of multiple slices. The weights were set such that data slices within each of the five domains contributed 1/5 of the overall score. Therefore, the contribution (effective weight) of each slice to the overall ToxPi model is the product of the domain weight by the slice weight given in Table 1. For example, the effective weight of the “Socioeconomic Status” slice is 3.3%, or 1/30 = (1/5) × (1/6). 

Using this weighting scheme, the complete data matrix was converted into a formatted model file using the free ToxPi GUI software (https://www.toxpi.org). The model file is available as Supplementary Materials (Table S1). For this application, the census-tract-wide ToxPi score for each slice was calculated by dividing the value for that census tract by the maximum across all tracts, yielding a slice score within the interval [0,1] for all 1090 census tracts. The linear(x) scaling option in ToxPi GUI was used, so values closer to the maximum would yield higher ToxPi scores, interpreted in this context as higher relative vulnerability (e.g., high levels of “PM2.5 Satellite” relative to other census tracts). Visually, the slice-wise scores translate into a distance from the origin (center) that is proportional to its normalized value. Census tracts with longer slices (i.e., farther from the origin) indicate greater vulnerability for that data element than tracts with shorter slices. 

Results were then uploaded to the HGBEnviroScreen ToxPi module. The module implements ToxPi as an ArcGIS frame within the HGBEnviroScreen dashboard (https://toxpi.org/gis/hgbenviroscreen/). The ArcGIS API for JavaScript was used to overlay ToxPi model images on custom ArcGIS maps. Model data were stored in an SQL database, which allows for complex SQL queries that can incorporate data from multiple models. ToxPi images are presented as inline SVG files, which significantly reduces the number of image requests to the server for large datasets and leads to faster rendering. In contrast to most screening tools, HGBEnviroScreen adds each model to the map as separate graphics layers so that results from multiple models can be displayed simultaneously. Users can tune the display of ToxPi model layers by adjusting both size and transparency. Complex queries can be generated by combining filters across models. Finally, the current map state (i.e., all visible settings) can be saved as a text file that can be shared with others or used as a means to continue analysis at a later date. This ability is key to promoting reproducible analyses.

A sensitivity analysis was carried out to identify factors driving tract scores. The analysis was implemented by ablating the influence of each domain, in turn, then recalculating new ToxPi scores for all tracts. Specifically, for each tract, the domain-specific score was replaced by overall median score and the rankings were recalculated. These new scores were compared to scores calculated using the original data to estimate the influence of each domain on a given tract’s overall score.

## 3. Results

### 3.1. Prioritization Across Census Tracts

**Figure 2:**
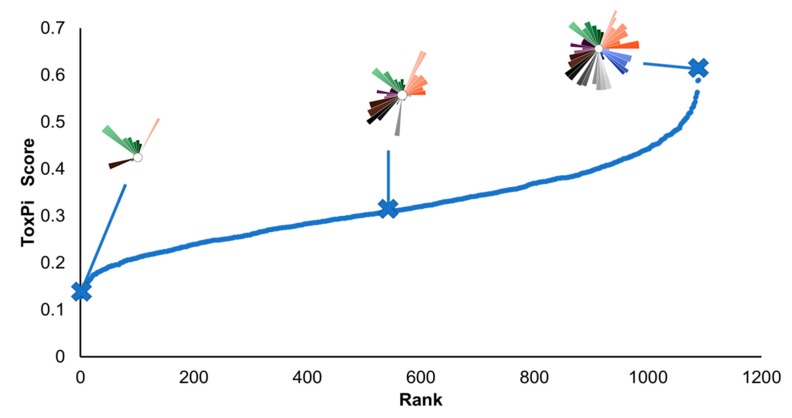
The overall distribution of Toxicological Prioritization Index (ToxPi) scores across all 1090 census tracts. Each dot in this rank plot is one tract. The profiles of the top (i.e., highest ToxPi score on vertical axis), median, and lowest tracts are shown along the distribution. ToxPi indicator colors are shown in Figure 1 (starting from the 3 o’clock position, going counterclockwise: orange = social vulnerability; green = baseline health; purple = environmental exposures and risks; grey = environmental sources; blue = flooding).

**Figure 3:**
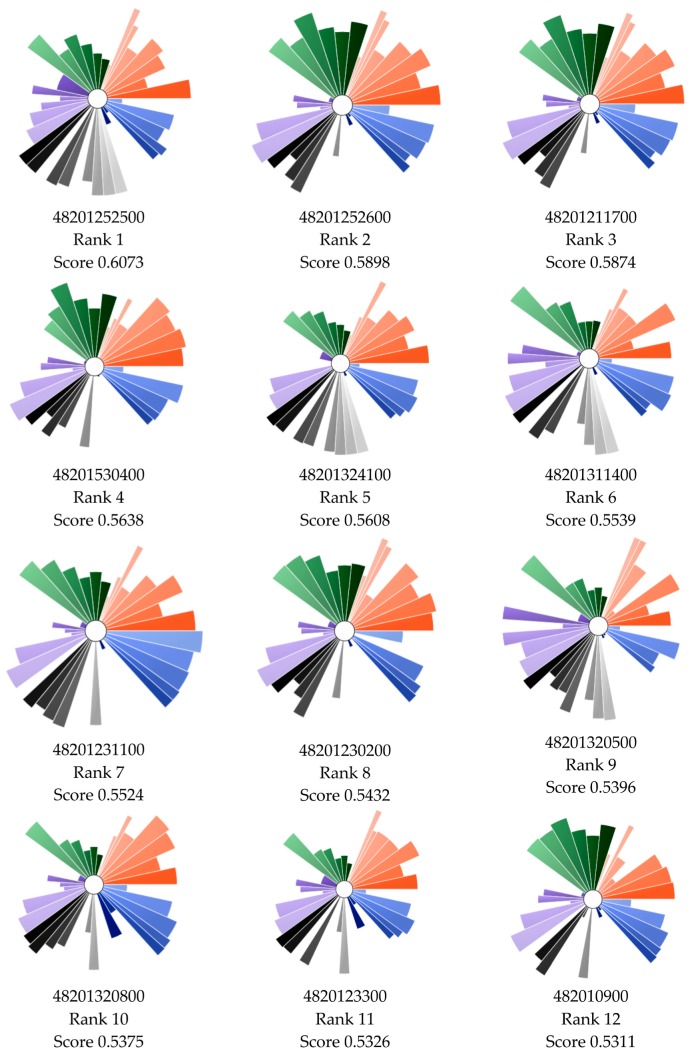
ToxPi visualizations of the top 10 ranking census tracts. ToxPi indicator colors are shown in Figure 1 (starting from the 3 o’clock position, going counterclockwise: orange = social vulnerability; green = baseline health; purple = environmental exposures and risks; grey = environmental sources; blue = flooding).

Figure 2 shows cumulative distribution of ToxPi scores for all 1090 census tracts. Each ToxPi shows not only the total ranking but also the contributors to the ranking by domain and indicator. Figure 3 shows the array of top 12 ranking ToxPis based on overall ToxPi score. As part of this project, we selected three CBOs representing neighborhoods which were among the top 5% most vulnerable census tracts in the HGB area based on their ToxPi score. ToxPis showing which domains and indicators are driving vulnerability in the census tract provide an important mechanism for communication with community groups. 

**Figure 4:**
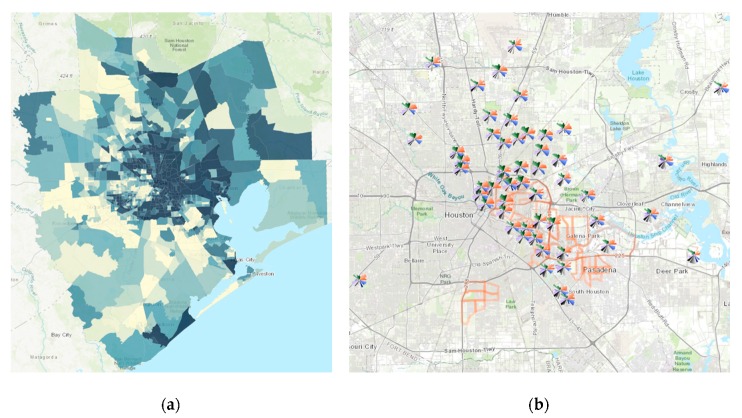
Map of spatial distribution of overall ToxPi scores across all 1090 census tracts (**a**). Overlay of ToxPi visualizations zoomed into the area containing the top 5% ToxPi scoring census tracts, along with census tracts in partner communities (orange outlines) for reference (**b**).

Figure 4a shows the ArcGIS visualization map for calculated overall ToxPi score, with colors indicating quintiles. The darker the rank score, the greater the vulnerability. Tracts having higher ToxPi scores do not appear randomly distributed. Spatially, the most vulnerable neighborhoods appear concentrated in East Houston where the concentrations of industrial facilities, low wealth, and minority populations are the greatest. Figure 4b shows the integrated result of ToxPi and GIS visualization, where ToxPi arrays are overlaid on the GIS map. This figure not only helps to pinpoint more vulnerable areas but also enhances the visualization to prioritize which domain can be of particular interest while working with each individual tract. It also allows CBOs to compare differences in vulnerability by indicator and domain for high-ranking versus lower-ranking neighborhoods.

### 3.2. Sensitivity Analysis: Primary Contributors to Vulnerability

**Figure 5:**
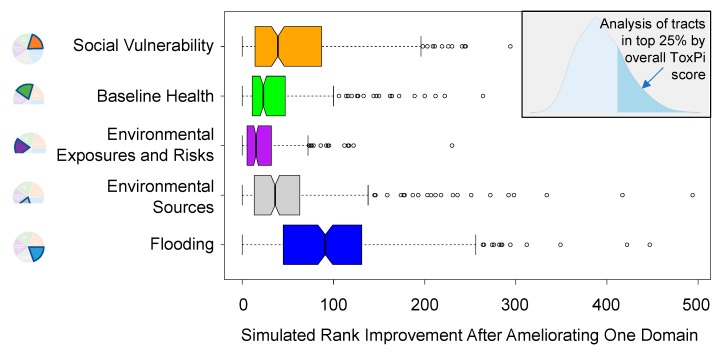
Sensitivity analysis results of simulated rank improvements after ameliorating vulnerabilities in a single domain. The histogram inset shows the overall distribution of ToxPi scores (one per tract), with the top 25th percentile of tracts shaded in the right tail. Among these census tracts, the rank improvement was calculated in our simulated experiments of addressing measures within a single domain, and the results across tracts are shown in boxplots for each domain.

The domain-wise sensitivity analysis can be used to determine which factors are driving ToxPi scores for each census tract. A change in overall ToxPi score can be used to prioritize areas that would most benefit from attention to particular domains. For example, Figure 5 shows the rank shift for the top 25th percentile of all tracts, after having improved scores for each domain back toward the median (i.e., ameliorated or improved metrics within a domain to the domain-wise median value across all tracts). In this analysis, improving flooding resilience had the greatest impact in the top quartile of census tracts, which is visible in the right panel of Figure 5 as high-magnitude rank changes for the “Flooding” domain. While the five domains differed in distribution of rank change (i.e., the magnitude of change across all census tracts in the top 25th percentile), the boxplot shows that each domain includes individual census tracts, the score of which may be improved by over 100 ranks. The results show the potential impact of addressing each domain for the communities of highest concern, as well as highlighting individual tracts that would most benefit from specific interventions.

**Figure 6:**
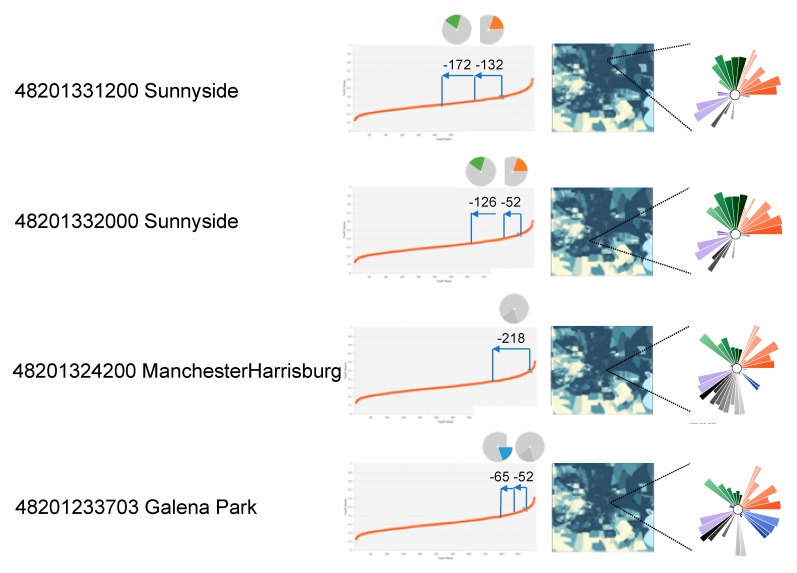
The ToxPi inset shows the original profile of tracts 48201331200, 48201332000, 48201324200, and 48201233703. The four rank plots show the effects on its original priority rank after ablating the effects of slices within either Social Vulnerability, Baseline Health, Environmental Sources, or Flood.

Alternatively, the sensitivity analysis can be used to estimate the impact of addressing a particular community. Figure 6 shows the results for several specific census tracts, where the results provide a way to address the question, “How would priority rankings change for a given neighborhood if we ameliorated factors within a domain?” Four tracts of interest—48201331200, 4201332000, 48201324200, and 48201233703—were used as an example. As seen in the profile in Figure 6, tracts 48201331200 and 4201332000 had relatively high concern for the domains of social vulnerability and baseline health (see Table 1), whereas the other two tracts (48201324200 and 48201233703) had relatively high values for environmental sources, and environmental sources and flooding, respectively. Ameliorating the effects from one domain could reduce the overall ranking of the census tract by at least 50 (and in some cases, more than 200), while addressing two domains could lead to rank reductions of at least 100. However, these results also show that different approaches would be needed in different census tracts to achieve the same results.

### 3.3. Case Example: Galena Park

One census tract within the Galena Park community (48201233300) ranks in the top 1% most vulnerable neighborhoods within the HGB region and is a good case example of how HGBEnviroScreen can be applied in a community-based setting. Galena Park is located east of the 610 freeway and north of the Ship Channel, with 90% of people living within a mile of an industrial facility [36]. This area was flooded heavily after Harvey, preventing the community from evacuating due to the lack of an evacuation route. Residents are predominantly Hispanic (some undocumented) and low to middle income. Routine chemical releases and elevated concentrations of air toxics are reported annually. Environmental Community Advocates of Galena Park (ECAGP) is one of the selected community-based organizations in Galena Park with whom we are partnering to develop environmental justice community action plans.

Currently, ECAGP is using data from the ToxPi assessment to create action plans to reduce the overall health risk identified through the various vulnerability indicators. In addition to ranking within the top 1% overall, Galena ranked among the top 5% for pollution sources, social vulnerability, and environmental exposures and risks domains in ToxPi. Consequently, ECAGP is prioritizing improving air quality in their community, making emergency procedure information available in Spanish language, advocating for membership on its local emergency planning committee, and addressing food desert issues in their community action plan. In collaboration with the University of Texas Environmental Law Clinic and Lone Star Legal Aid, we are working to assist the community in understanding these vulnerability indices in the context of historical events that may have contributed to the community’s risk profile. Ultimately, the goal is to identify whether there may be legal avenues that could be employed to mitigate potential sources of risk. Additional goals include working with community partners, stakeholders, and emergency plan responders to increase environmental awareness, to improve environmental and health quality, and to build resilience in the community.

## 4. Discussion

While federal policies and an executive order by President Clinton to address environmental disparities in low socioeconomic communities have yielded improvements in environmental conditions, environmental disparities remain. In addition to being in proximity to a high concentration of industrial facilities, EJ communities continue to live in urban areas plagued with high lead concentrations and hazardous air pollution at concentrations frequently exceeding federal regulatory standards [37,38,39,40,41]. Moreover, African-American and Hispanic communities are often racially segregated; suffer from food insecurity; and lack proximity to services such as health care facilities, good schools, grocery stores, and green spaces. In addition to social and material deprivations, they may be further burdened with traffic density, lack of affordable housing, and crime, which can increase exposure to physical environments and have lasting negative impacts on community health [42]. As Brulle and Pellow accurately state, “the stark spatial distribution of environmental disamenities in society also produce health disparities [37]”. Thus, these communities not only experience cumulative impacts from multiple chemical exposures, but vulnerabilities from social deprivation and other factors must be taken into consideration if mitigation of health disparities in EJ communities is to be achieved. 

Recent events ranging from frequent flooding and hurricanes, such as Harvey, to large-scale industrial accidents, such as the petrochemical fire at the Intercontinental Terminal Company facility, have demonstrated the unique and multifaceted environmental vulnerabilities that face the HGB area. This region represents the potential for a “perfect storm”, in the confluence of the high incidence of natural disasters, the large presence of hazardous chemicals, and the presence of many underserved EJ communities. Moreover, these EJ communities are generally at greater risk because of additional vulnerabilities related to social and economic disadvantages, poorer infrastructure and support services, and proximity to sources of risk. This convergence of vulnerabilities highlights the critical need for data and approaches that characterize and integrate multiple factors in a manner that can inform prioritization and decision-making. 

Existing EJ mapping tools have made progress in providing communities with actionable information in order to advocate for improvements to their environment. However, these tools usually require a tradeoff between aggregation of multiple metrics into a single index and the ability to explore individual indicators. For instance, CalEnviroScreen and MD EJSCREEN produce overall EJ scores at the census tract level [6,43], whereas the EPA’s EJSCREEN provides a multitude of indicators without an overall score [7]. Moreover, U.S.-wide tools such as the EPA’s EJSCREEN do not address local issues that may be unique to a particular location.

In developing HGBEnviroScreen, we aimed to address these limitations by combining GIS-based mapping, the creation of an overall index, and advanced visualization techniques to enable disaggregation of index components. This approach was enabled by merging the ToxPi methodology with ArcGIS. The result is the ability to take advantage of multistream data while also ranking, prioritizing, and visualizing the richness of the information. Critically, the HGBEnviroscreen interface maintains the ability to disaggregate overall scores into domain-specific visualizations. The ability to layer domain models (*Layers* menu option) and create complex filters (*Filters* menu option) gives users an easy way to ask complex questions about areas of concern and their geographic distribution.

An additional advantage of this approach is that it enables communities to identify key drivers of their increased vulnerability, thereby providing a data-driven approach to advocating for change. For instance, in our analysis, some highly vulnerable census tracts could change their ranking by 10% or 20% by addressing just one key area of vulnerability. 

Data visualization and mapping are powerful tools for communicating scientific data and the concept of cumulative impacts to the average citizen. During community group meetings presenting ToxPi results, residents were able to see and compare ToxPi visualizations and rank scores for their neighborhood relative to communities in close proximity and similar in nature as well as to neighborhoods far away or with different socioeconomic composition. In doing so, residents saw the stark contrasts in indicator data and ranking especially when compared to the other 1089 census tracts in the eight-county HGB region. Their results frequently elicited surprise or disappointment in the level of vulnerability reflected in the data but were a strong motivating factor to address these drivers and improve quality of life. Frequently, residents and community organizations lack data or access to data that could provide empirical evidence that may form the foundation for environmental policy. ToxPi and HGBEnviroscreen remove this barrier and make the data contained in this tool both accessible and understandable to the layperson.

Several local communities are already utilizing HGBEnviroScreen in their advocacy efforts. For instance, Pleasantville Neighborhood CBO, Achieving Community Tasks Successfully (ACTS), noted that ToxPi indicator layers (such as damage after Hurricane Harvey and percentage of low- to middle-income neighborhoods that had FEMA assistance applications served) demonstrated a disparity in services to their community, thus prompting advocacy efforts with City of Houston officials to address this unacceptable inequity. In another example, based on social vulnerability, pollution sources, and health domain scores, the 5th Ward community is working to address blood lead levels among children and housing exposures. Other communities where air pollution appears to be a major driver have been participating in additional data collection efforts using air monitors mounted within their neighborhoods. Their goal is to obtain local data on regulatory exceedances and their potential health impacts. Several selected CBOs are also addressing flooding and air pollution issues using mapping to determine where to install rain gardens and plant trees in collaboration with Trees for Houston and other nonprofit organizations. Additionally, understanding spatial differences in asthma prevalence, cardiac arrests, and life expectancy in combination with environmental exposure data can be critical input for communities working with city and county public health and pollution control departments to target where intervention programs are needed most. Finally, in order better understand air pollution in their neighborhood and its impacts on health and education, several communities are developing plans for low-cost residential air monitoring networks and citizen science advocacy.

## 5. Conclusions

We have developed an online EJ mapping tool, HGBEnviroScreen, that provides three key services to communities and local decision-makers:

- Incorporating data on key, multifaceted vulnerabilities, including both common EJ concerns such as social vulnerability and air pollution, as well as HGB-specific issues such as flooding and proximity to large numbers of industrial facilities;
- Aggregating five key domains of vulnerability into an overall ToxPi score at the census tract level, thereby facilitating identification and prioritization of highly vulnerable communities;
- Visualizing both the overall ToxPi score as well as the relative contribution of its component indicators, so as to enable communities to target advocacy and interventions that create the greatest value in terms of reducing vulnerability.

Our community partners are already taking these data and results and using them in development of community action plans. We anticipate that HGBEnviroScreen will continue to empower communities to advocate for EJ, with the ultimate goal of reducing environmental disparities and improving their health and well-being.

## Figures and Tables

**Table 1:** Indicators and the domains of vulnerability incorporated into HGBEnviroScreen.

Domain (% Weight of Overall Score) Indicator (Fractional Weight within Domain)	Indicator Unit for Each Census Tract	Ref.
Social Vulnerability (20%)		
Socioeconomic status (1/6)	% ile index	[23]
Household composition and disability (1/6)	% ile index	[23]
Minority status and language (1/6)	% ile index	[23]
Housing and transportation (1/6)	% ile index	[23]
Without health insurance (1/6)	% of population	[23]
Modified food retail environment index (1/18)	100—MFREI Score ^1^	[24]
Food desert low access (1/18)	Sum of two low access flags	[25]
Low food security (1/18)	% of population	[26]
Baseline Health (20%)		
Childhood asthma (1/6)	% crude prevalence	[17]
Stroke (1/6)	% crude prevalence	[17]
Chronic obstructive pulmonary disease (1/6)	% crude prevalence	[17]
Coronary heart disease (1/6)	% crude prevalence	[17]
Life expectancy (1/6)	100—Life exp in years ^1^	[18]
Proximity to hospitals (1/6)	100—# within 5 km ^1^	[27]
Environmental Exposures and Risks (20%)		
Risk-screening environmental indicators (1/3)	Aggregated 2015–2017 Score	[28]
National Air Toxics Assessment (NATA) Cancer (1/9)	Cancer risk	[20]
NATA Respiratory Tract (1/9)	Hazard index	[20]
NATA Reproductive (1/9)	Hazard index	[20]
PM2.5 Community Multiscale Air Quality (CMAQ) (1/6)	μg/m^3^	[21]
PM2.5 Satellite (1/6)	μg/m^3^	[22]
Environmental Sources (20%)		
Major roads (1/10)	% ile based on # within 1 km	[29]
Cement batch plants (1/10)	% ile based on # within 1 km	[30]
Metal recyclers (1/10)	% ile based on # within 1 km	[30]
Petrochemical and oil refineries (1/10)	% ile based on # within 1 km	[19]
Power plants (1/10)	% ile based on # within 1 km	[31]
Superfund sites (1/10)	% ile based on # in census tract	[32]
Leaking petroleum storage tanks (1/10)	% ile based on # in census tract	[32]
Facilities with risk management plans (RMP) (1/10)	% ile based on # in census tract	[33]
Accident events reported in RMP (1/10)	% ile based on # in census tract	[33]
Shelter-in-place events reported in RMP (1/10)	% ile based on # in census tract	[33]
Flooding (20%)		
100 year flood plain (1/6)	% ile based on fraction of area	[29]
500 year flood plain (1/6)	% ile based on fraction of area	[29]
Harvey damage assessment “Affected” (1/12)	% ile based on # in census tract	[34]
Harvey damage assessment “Minimal” (1/12)	% ile based on # in census tract	[34]
Harvey damage assessment “Major” (1/6)	% ile based on # in census tract	[34]
Harvey damage assessment “Destroyed” (1/6)	% ile based on # in census tract	[34]
Families filing Harvey damage claims (1/6)	% of population	[35]

^1^ 100 minus the indicator was used so that higher values represent more vulnerability.

## Supplementary Materials

The following are available online at https://www.mdpi.com/1660-4601/17/4/1130/s1: ToxPi_InputFile.csv.

## References

[1] United Church of Christ Commission for Racial Justice (1987). Toxic Wastes and Race in the United States: A National Report on the Racial and Socio-Economic Characteristics of Communities with Hazardous Waste Sites.

[2] Bullard R.D., Wright B.H. (1993). Environmental justice for all: Community perspectives on health and research needs. Toxicol. Ind. Health.

[3] Bethel H.L., Sexton K., Linder S., Abramson S., Bondy M., Fraser M., Ward J. (2006). A Closer Look at Air Pollution in Houston: Identifying Priority Health Risks.

[4] Vojnovic I. (2003). Governance in Houston: Growth Theories and Urban Pressures. J. Urban Aff..

[5] Wilson W.J. (2008). The Political and Economic Forces Shaping Concentrated Poverty. Political Sci. Q..

[6] Faust J., Laura A., Komal B., Vanessa G., Julian L., Shankar P., Rose S., Andrew S., Robbie W., Walker W. (2017). Update to the California Communities Environmental Health Screening Tool CalEnviroScreen 3.0.

[7] US EPA (2017). EJSCREEN Technical Document.

[8] Rowangould D., Rowangould G., Craft E., Niemeier D. (2018). Validating and Refining EPA’s Traffic Exposure Screening Measure. Int. J. Environ. Res. Public Health.

[9] Marvel S.W., To K., Grimm F.A., Wright F.A., Rusyn I., Reif D.M. (2018). ToxPi Graphical User Interface 2.0: Dynamic exploration, visualization, and sharing of integrated data models. BMC Bioinform..

[10] Reif D.M., Sypa M., Lock E.F., Wright F.A., Wilson A., Cathey T., Judson R.R., Rusyn I. (2013). ToxPi GUI: An interactive visualization tool for transparent integration of data from diverse sources of evidence. Bioinformatics.

[11] Chiu W.A., Guyton K.Z., Martin M.T., Reif D.M., Rusyn I. (2018). Use of high-throughput in vitro toxicity screening data in cancer hazard evaluations by IARC Monograph Working Groups. ALTEX.

[12] National Academies of Sciences, Engineering, and Medicine (2017). Using 21st Century Science to Improve Risk-Related Evaluations.

[13] Loomis D., Guyton K., Grosse Y., El Ghissasi F., Bouvard V., Benbrahim-Tallaa L., Guha N., Mattock H., Straif K. (2015). Carcinogenicity of lindane, DDT, and 2,4-dichlorophenoxyacetic acid. Lancet Oncol..

[14] Zirogiannis N., Hollingsworth A.J., Konisky D.M. (2018). Understanding Excess Emissions from Industrial Facilities: Evidence from Texas. Environ. Sci. Technol..

[15] Tabuchi H. (2017). High Levels of Carcinogen Found in Houston Area After Harvey. The New York Times.

[16] Flanagan B.E., Hallisey E.J., Adams E., Lavery A. (2018). Measuring Community Vulnerability to Natural and Anthropogenic Hazards: The Centers for Disease Control and Prevention’s Social Vulnerability Index. Agency Toxic Subst. Dis. Regist..

[17] CDC (2018). 500 Cities: Local Data for Better Health, 2017 Release.

[18] USALEEP (2018). U.S. Small-Area Life Expectancy Estimates Project.

[19] TRI, EPA (2019). TRI Basic Data Files: Calendar Years 1987–2017.

[20] US EPA (2017). Chemical Concentrations, Exposures, Health Risks by Census Tract from National Scale Air Toxics Assessment (NATA).

[21] US EPA Community Multiscale Air Quality Modeling System: RSIG-Related Downloadable Data Files. https://www.epa.gov/hesc/rsig-related-downloadable-data-files.

[22] Di Q., Amini H., Shi L., Kloog I., Silvern R., Kelly J., Sabath M.B., Choirat C., Koutrakis P., Lyapustin A. (2019). An ensemble-based model of PM2.5 concentration across the contiguous United States with high spatiotemporal resolution. Environ. Int..

[23] CDC Social Vulnerability Index 2016 Database Texas. https://svi.cdc.gov/data-and-tools-download.html.

[24] CDC Census Tract Level State Maps of the Modified Retail Food Environment Index (mRFEI). https://www.cdc.gov/obesity/downloads/census-tract-level-state-maps-mrfei_TAG508.pdf.

[25] USDA Food Access Research Atlas Data. https://www.ers.usda.gov/data-products/food-access-research-atlas/download-the-data/.

[26] Gundersen C., Dewey A., Crumbaugh A., Kato M., Engelhard E. (2018). Map the Meal Gap 2018: A Report on County and Congressional District Food Insecurity and County Food Cost in the United States in 2016.

[27] Texas DSHS Texas Acute and Psychiatric Hospitals as of July 2019. https://dshs.texas.gov/chs/hosp/Hosplis2019.pdf.

[28] US EPA Risk-Screening Environmental Indicators (RSEI) Model. https://www.epa.gov/rsei.

[29] Houston-Galveston Area Council GIS Datasets. http://www.h-gac.com/gis-applications-and-data/datasets.aspx.

[30] Lewis G., City of Houston (2019). Concrete Crusher, Cement Batch Processors, Metal Recycler Data.

[31] U.S. Energy Information Administration Power Plants Shapefile. https://www.eia.gov/maps/map_data/PowerPlants_US_EIA.zip.

[32] TCEQ TCEQ GIS Data. https://www.tceq.texas.gov/gis/download-tceq-gis-data.

[33] Houston Chronical The Right-to-Know Network. http://www.rtk.net/.

[34] Homeland Infrastructure Foundation-Level Data (HIFLD) Subcommittee FEMA Modeled Building Damage Assessments Harvey 20170829. https://respond-harvey-geoplatform.opendata.arcgis.com/datasets/fema-modeled-building-damage-assessments-harvey-20170829/geoservice.

[35] Harris County Long Term Recovery Committee Data Workgroup (2018). Coordinated Assistance Network (CAN) Data System.

[36] UCS (2016). Houston Chemical Facilities Put Vulnerable Communities in Double Jeopardy.

[37] Brulle R.J., Pellow D.N. (2006). Environmental justice: Human health and environmental inequalities. Annu. Rev. Public Health.

[38] Miranda M.L., Edwards S.E., Keating M.H., Paul C.J. (2011). Making the environmental justice grade: The relative burden of air pollution exposure in the United States. Int. J. Environ. Res. Public Health.

[39] Tessum C.W., Apte J.S., Goodkind A.L., Muller N.Z., Mullins K.A., Paolella D.A., Polasky S., Springer N.P., Thakrar S.K., Marshall J.D. (2019). Inequity in consumption of goods and services adds to racial-ethnic disparities in air pollution exposure. Proc. Natl. Acad. Sci. USA.

[40] US EPA (1992). Environmental Equity Reducing Risk for All Communities.

[41] Woo B., Kravitz-Wirtz N., Sass V., Crowder K., Teixeira S., Takeuchi D.T. (2019). Residential Segregation and Racial/Ethnic Disparities in Ambient Air Pollution. Race Soc. Probl..

[42] Morello-Frosch R., Pastor M., Porras C., Sadd J. (2002). Environmental justice and regional inequality in southern California: Implication for future research. Environ. Health Perspect..

[43] Driver A., Mehdizadeh C., Bara-Garcia S., Bodenreider C., Lewis J., Wilson S. (2019). Utilization of the Maryland Environmental Justice Screening Tool: A Bladensburg, Maryland Case Study. Int. J. Environ. Res. Public Health.

